# Tuberculosis Infectiousness and Host Susceptibility

**DOI:** 10.1093/infdis/jix361

**Published:** 2017-11-03

**Authors:** Richard D Turner, Christopher Chiu, Gavin J Churchyard, Hanif Esmail, David M Lewinsohn, Neel R Gandhi, Kevin P Fennelly

**Affiliations:** 1 Department of Respiratory Medicine, King’s College Hospital;; 2 Section of Infectious Diseases & Immunity, Imperial College London, United Kingdom;; 3 Aurum Institute and; 4 School of Public Health, University of Witwatersrand, Johannesburg, South Africa;; 5 Radcliffe Department of Medicine, University of Oxford, United Kingdom;; 6 Wellcome Center for Infectious Diseases Research in Africa, University of Cape Town, South Africa;; 7 Department of Molecular Microbiology and Immunology, Oregon Health & Science University, Portland;; 8 School of Medicine and Rollins School of Public Health, Emory University, Atlanta, Georgia;; 9 Pulmonary Clinical Medicine Section, Cardiovascular Pulmonary Branch, Division of Intramural Research, National Heart, Lung, and Blood Institute, National Institutes of Health, Bethesda, Maryland

**Keywords:** Tuberculosis, transmission, infectiousness, susceptibility

## Abstract

The transmission of tuberculosis is complex. Necessary factors include a source case with respiratory disease that has developed sufficiently for *Mycobacterium tuberculosis* to be present in the airways. Viable bacilli must then be released as an aerosol via the respiratory tract of the source case. This is presumed to occur predominantly by coughing but may also happen by other means. Airborne bacilli must be capable of surviving in the external environment before inhalation into a new potential host—steps influenced by ambient conditions and crowding and by *M. tuberculosis* itself. Innate and adaptive host defenses will then influence whether new infection results; a process that is difficult to study owing to a paucity of animal models and an inability to measure infection directly. This review offers an overview of these steps and highlights the many gaps in knowledge that remain.

The transmission of any infectious disease requires a source, a susceptible new potential host, and passage of the pathogen, by direct contact, by indirect contact (eg, by fomites or an environmental medium, such as water), or via the air. Tuberculosis is the archetype of airborne-transmitted infectious diseases, with *Mycobacterium tuberculosis* serving as the causative agent [1]. For human tuberculosis, the source of the majority of new infections is other humans with pulmonary disease. Zoonotic tuberculosis, largely resulting from *Mycobacterium bovis* from cattle and their food products, is important in some contexts but accounts for only around 1.4% of cases of human tuberculosis overall [2].

Therefore, for the majority of cases, *M. tuberculosis* must exit the respiratory tract of the source case, survive the rigors of aerosolization and desiccation in the outside environment, be inhaled into the lung of the new potential host, and evade immune defenses to cause new infection (Figure 1). This overview presents what is known of these processes of tuberculosis transmission, and highlights where gaps in knowledge remain (Figure 2). 

**Figure 1.**
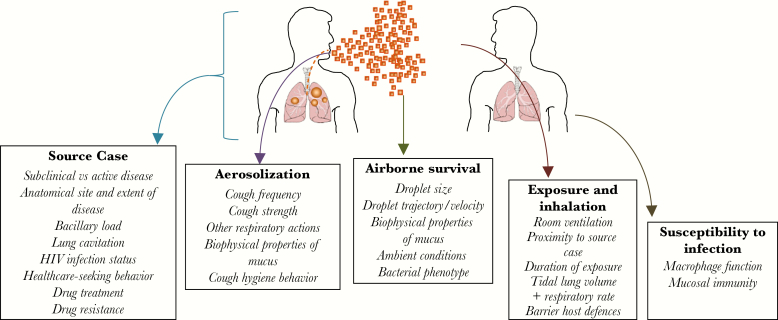
Theoretical scheme illustrating some steps and variables important for the transmission of tuberculosis. Abbreviation: HIV, human immunodeficiency virus.

**Figure 2.**
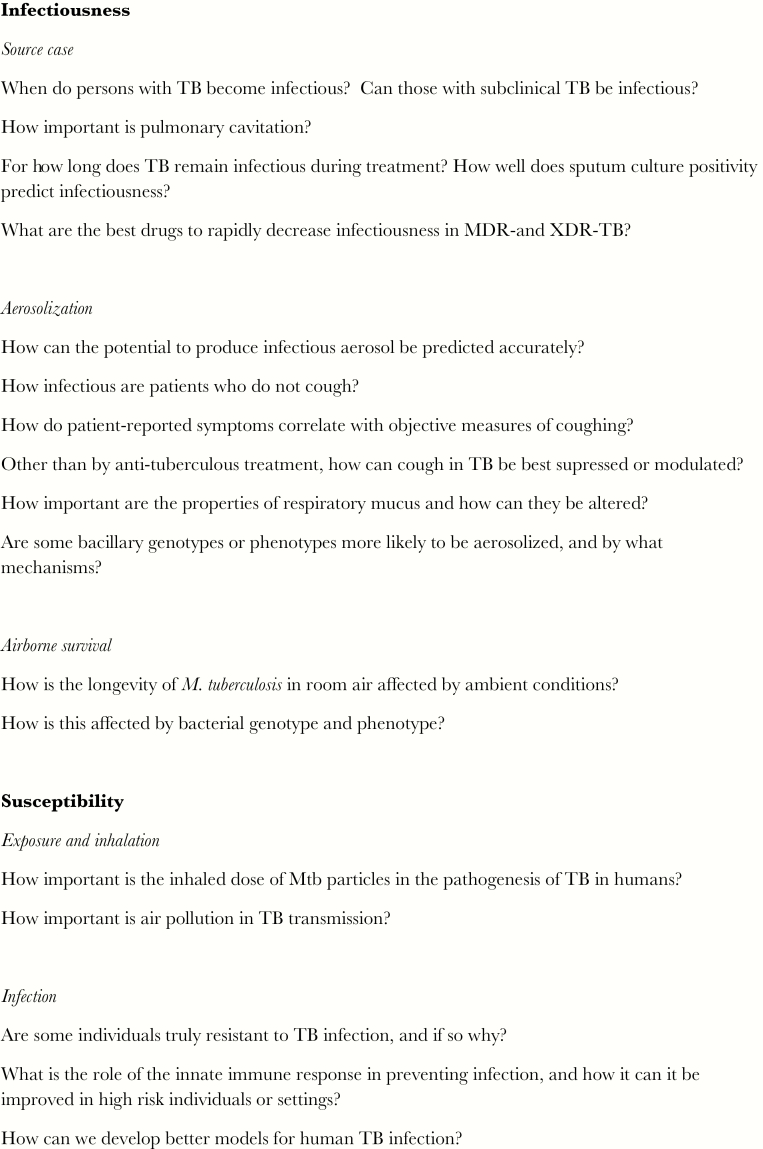
Key unanswered questions and research priorities.

## INFECTIOUSNESS

### Source Case

Tuberculosis has often been considered as distinct binary states of infection (asymptomatic, noninfectious with effective bacillary control) and disease (symptomatic, potentially infectious with failure of bacillary control). However, this is probably an oversimplification, which may be hindering efforts for tuberculosis control [3, 4]. The pathology, presence of culturable bacilli, and symptoms do not appear simultaneously but likely develop over time, perhaps intermittently, with disease present prior to symptoms [5].

The development of an acquired cell-mediated immune response to *M. tuberculosis* 6–8 weeks following initial infection and dissemination facilitates the effective control of *M. tuberculosis* within granulomata [6]. This is manifested by positive results of a tuberculin skin test (TST) or interferon γ–release assay (IGRA). The true extent of quiescence of this state of so-called latent infection is poorly understood. Similarly, little is known about the point of transition from this state to tuberculosis [4].

The process of failure to contain bacilli within granulomata has recently been demonstrated in a nonhuman primate model, resulting in pulmonary consolidation and bronchogenic spread of cellular infiltrate [7]. In human autopsy studies, a similar pneumonic infiltration has been observed as the first pathological sign of pulmonary disease. The initially small number of mycobacteria increases substantially as necrosis develops [8]. High-resolution imaging in clinical studies has also identified lung consolidation in some asymptomatic persons with a conventional diagnosis of latent *M. tuberculosis* infection, correlating with a greater risk of symptomatic disease [5].

Development of pathology likely precedes shedding of bacilli into sputum and symptoms. Attempts have been made to estimate the duration of sputum positivity before clinical presentation, through community surveys which compare the prevalence of diagnosed and undiagnosed culture-positive tuberculosis [9]. The extent of transmission by patients with subclinical tuberculosis is currently unknown. However, it is a critically important parameter to understand; modeling of transmission during this early stage of tuberculosis indicates that detecting subclinical disease has a large effect on the 10-year incidence of tuberculosis [10].

Subclinical disease is also important to consider because of the potential to lead to the development (and onward transmission) of drug-resistant bacterial strains following inadvertent undertreatment with tuberculosis chemoprophylaxis. In particular, isoniazid monoresistance may be associated with isoniazid preventive therapy [11].

Key events in the pathogenesis of pulmonary tuberculosis are necrosis at the site of pneumonia and cavitation [12]. Cavitation results in a step up in bacillary load, owing to an increased concentration of oxygen which favors bacillary growth, and an environment preventing immunological control [13].

Cavity formation is linked to immunocompetence, which is notably reduced in human immunodeficiency virus (HIV) coinfection, transplant recipients, and elderly individuals [14, 15]. The impact of sex, ethnicity, and infecting strain on cavitation is less certain. In addition to the impact of general immunity, the local environment of granulomata probably strongly influences cavity progression [16]. The modulation or prevention of cavity formation could have significant effects on tuberculosis transmission, through reduced bacterial growth and access to the airways for aerosolization.

Progression of disease once precipitated is unlikely to be linear. Spontaneous regression and healing (with subsequent fibrosis and calcification) even of symptomatic, cavitary disease is long recognized [17]. Similarly, apical fibrocalcific scarring in the absence of previous history of disease is frequently observed, although with an increased risk of subsequent progression and clinical tuberculosis [18].

Extrapulmonary tuberculosis represents a dead end for the life cycle of *M. tuberculosis*, yet determinants of the anatomical site of disease are poorly understood. These forms of disease represent around 15% of all global tuberculosis notifications [19], although many cases of extrapulmonary tuberculosis probably go unreported because of the difficulty of diagnosis, compared with pulmonary disease. Conversely, there may be unrecognized pulmonary tuberculosis coexisting with extrapulmonary disease [20]. Nevertheless, several broad epidemiological variables have been associated with a tendency to develop nonpulmonary rather than pulmonary tuberculosis, such as female sex, time since infection, and, possibly, mycobacterial lineage [20, 21].

The role of HIV coinfection on the infectiousness of patients with tuberculosis has been debated [22]. The presence of smear-positive sputum or cavitary disease seems to be associated with *M. tuberculosis* infection in contacts regardless of the HIV status of the index case [23]. However, HIV seems to reduce infectiousness overall, owing to an association with less cavitary disease, lower sputum bacillary loads, a reduced tendency for prolonged illness [24], and, presumably, more-general debility in advanced disease, which reduces opportunities for social mixing.

Infectiousness ceases with appropriate therapy long before pulmonary tuberculosis is cured. A much-reported conclusion by Rouillon et al was that transmission ceases within 2 weeks after initiating effective antituberculous treatment [25]. However, the cited epidemiological studies were limited by using as their outcomes the rates of *M. tuberculosis* infection among household contacts of individuals who had been hospitalized for treatment. It is likely that most susceptible contacts in those homes were exposed and infected before the hospital admission of the index case, or even infected outside the household. The evidence from these data of a 2-week period of infectiousness following initiation of effective treatment is therefore limited [26].

Another approach suggested that *M. tuberculosis* may be viable after 6–7 weeks of treatment, based on the inoculation of bacilli from sputum samples into guinea pigs [27]. However, experimental studies using natural airborne infection of guinea pigs by persons with tuberculosis have suggested that transmission may stop within 24–48 hours [28]. Limited data in multidrug-resistant tuberculosis (MDR-TB) indicate that growth in cultures of cough aerosol specimens decreases exponentially over 3–21 days after the initiation of effective treatment [29].

The efficacy and availability of treatment are increasing problems with the emergence of drug resistance, and they are priorities for tuberculosis control. Not only do individuals with drug-resistant tuberculosis probably remain infectious for longer than those with drug-susceptible disease during treatment, treating secondary cases is more complicated and costly. The problem might in part be mitigated by a relative reduction in the fitness of drug-resistant strains of *M. tuberculosis*. The data on this are conflicting, although a recent large prospective study in Peru estimated a reduction in transmission to household contacts of about 50% by source cases who had MDR-TB as compared to those who had fully drug-susceptible disease [30]. This finding may not be universal to different human populations and MDR *M. tuberculosis* strains [31].

### Aerosolization

Although individuals with cavitary disease and a high bacillary burden are more likely to release *M. tuberculosis* into the environment, it is clear from epidemiological studies that not all of these patients are infectious. This has been confirmed by studies using a novel cough aerosol sampling system; only a proportion of patients with sputum smear–positive tuberculosis generated droplets containing culturable bacilli [32]. Cough aerosol specimens yielding high culture counts were the only predictor of new infection among exposed contacts [33]. Bacillary load and salivary sputum are predictors of cough aerosol production, but there remains considerable unexplained variability in infectiousness among source cases [32].

Droplet particles are released from the respiratory tract during all respiratory activities. Relevant variables for droplet infectiousness include their number, initial velocity and trajectory, size, and composition [34]. Although there have been very few studies involving patients with tuberculosis, in healthy volunteers greater numbers of particles are released during coughing than during tidal breathing or when talking [35]. Singing [36] and blowing on wind instruments [37] may also produce large numbers of respiratory particles. High shear forces are likely to be helpful for releasing bacteria from cavities and areas of bronchial obstruction and for overcoming mucus viscosity, to allow droplets to tear away from the airway lumen. Coughing is associated with more force and higher airflow velocities than breathing or talking [38] and is also relevant for the rapid spread of airborne bacilli away from the patient in an enclosed space.

Respiratory particles from coughing are probably of higher median diameter than those released during talking and tidal breathing, in which a larger proportion have a diameter of <1 µm [35]. Although influenza virus is found in exhaled breath [39], the larger size of *M. tuberculosis* (>2 µm) probably precludes its presence in aerosol particles with a diameter of <1 µm. Because viable *M. tuberculosis* has yet to be demonstrated in breath, the apparent increased release of particles during tidal breathing by patients with tuberculosis as compared to healthy controls is of unclear significance [40].

Although coughing is probably more important than other respiratory activities for infectiousness in tuberculosis [34], this has been rarely studied. A recent study has confirmed that cough frequency is associated with infection in household contacts [41]. Cough frequency seems to be associated with sputum bacillary load [42] and is possibly associated with radiographic extent of disease [43]. However, other causes of the wide variation of cough frequency in patients with tuberculosis remain largely obscure [34]. Cough strength has been studied even less frequently. One study did not find an association of new infections with cough peak flow rates or observational assessment of cough severity [33]. However, another study found subjective assessment of cough severity to be associated with more transmission [44].

The nature of airway mucus presumably affects infectiousness in at least 2 ways. The rheological properties of secretions, including viscosity, elasticity, and cohesivity, predict the potential for aerosolization [45]. We are not aware of studies of how mucus and other components of secretions might protect bacilli during aerial transit. Mucus properties vary with disease and with other factors, such as smoking [46], although this is another topic for which additional research is needed. Furthermore, pharmacologically altering the properties of mucus could alter the transmissibility of tuberculosis by individuals with difficult-to-treat (eg, extensively drug-resistant) disease.

Lung capacity, the anatomy of the lower and upper airway, and the position of the tongue during coughing probably all affect aerosol production, although all appear to have been little investigated [34]. In patients with cystic fibrosis, lung function is associated with the aerosolization of *Pseudomonas aeruginosa* when coughing [47], but this has not been studied in patients with tuberculosis. Behavioral aspects, such as cough hygiene, potentially limit the release of infectious aerosols; for example, surgical masks worn by patients with tuberculosis have been shown to reduce the risk of transmission by >50% [48]. Behavioral change clearly depends on conscious awareness of coughing, which seems to be variable [43].

Estimates have been made of rates of release of infectious particles in tuberculosis, mainly using the guinea pig air-sampling model [49]. The smallest inhaled dose required for infection, presumed (but not definitively proven) to correspond to a single bacillus, has been termed a quantum. Release of quanta appears highly variable among patients with smear positive disease; one particularly infectious individual in one study exceeded 200 quanta/hour, although the median rate in patients causing new infection was 12 quanta/hour (interquartile range, 4.3–39.0 quanta/hour) [50]. Easier and more-accurate identification of the most infectious individuals would be of significant value to enable better targeting of infection control measures and contact tracing efforts.

### Airborne Survival

Life outside the human host presents a number of potential challenges to the survival of *M. tuberculosis.* The time during which bacilli remain capable of causing infection after release into the environment is highly relevant for transmission, yet there is a basic lack of knowledge of the longevity of *M. tuberculosis* in open air. Ambient conditions, including ultraviolet and ionizing radiation, are relevant, and germicidal irradiation is thus used for infection control [51, 52]. Features of the organism are also important; it has recently been recognized that phenotypic changes may occur in *M. tuberculosis* in association with the airborne phase of its life cycle [53].

Investigations of outbreaks of tuberculosis in congregate settings and infection of guinea pigs exposed to air ventilated from remote wards housing source patients [28] have highlighted the ability of *M. tuberculosis* to remain viable and be transported through air in indoor spaces. Novel approaches are required to learn more about the aerobiology of *M. tuberculosis* [49].

## SUSCEPTIBILITY

### Exposure and Inhalation

Mathematical modeling has informed the variables determining the transmission of tuberculosis indoors, especially in demonstrating the strong effects of poor ventilation and overcrowding in increasing exposure of occupants to higher concentrations of infectious particles. The effective contact rate is determined by the room ventilation rate (calculated as the number of air exchanges per hour), room volume, occupation density, and duration of exposure [54]. It can be reduced by increasing the room ventilation or volume or reducing the occupation density [54].

The number of air exchanges per hour may be increased through natural, mechanical, or mixed methods of ventilation [55]. Mechanical ventilation can provide a constant fixed rate of air exchange, with control of the airflow direction independent of ambient conditions, but it is relatively costly to install, requires expertise to maintain, and, in some high-burden settings, is inadequate [56]. Natural ventilation can be achieved by opening doors and windows (which can increase the ventilation rate to a value such as 28 air changes/hour), particularly in rooms with high ceilings and large windows (which can yield an increase of up to 40 air changes/hour) [57]. Determinants of natural ventilation include wind speed, window size, and cross ventilation [57]. Natural ventilation as compared to mechanical ventilation is cheaper and easier to maintain, and it can potentially be used in a large range of settings. However, the acceptability and effectiveness of natural ventilation is affected by weather and climate conditions, and mechanical ventilation might be better suited for providing the optimum design of positive pressure ventilation in staff areas and negative pressure ventilation in patient areas.

Higher inhaled doses of infectious particles probably more likely result in tuberculosis [58]. A determinant of inhaled dose is proximity of contact to the source case; individuals sharing a bed with people with tuberculosis have the greatest risk of becoming infected [59]. The intensity of exposure over time is also a surrogate for inhaled dose. Among South African gold miners, the estimated annual risk of infection is 20% per year; around 89% are infected with *M. tuberculosis* after approximately 20 years in the occupation [60]. Highly exposed healthcare workers in South Africa have a 2–4-fold greater risk of *M. tuberculosis* infection, compared with medical students with low exposure [61].

Host variables are relevant for the inhalation of infectious particles. Respiratory rate and tidal volume increase the risk of infection [54], whereas respirator masks (when used) probably help prevent tuberculosis [62]. Barrier host defenses, which limit entry of *M. tuberculosis* into the distal lung, are also clearly important and include a functioning respiratory epithelium and an effective mucociliary escalator [46, 63]. Smoking is an important epidemiological risk factor for tuberculosis and probably exerts at least some of its deleterious effect through an impact on these basic respiratory defenses [64]. Air pollution may also have an important impact [65].

### Infection

At present, the mechanisms underlying resistance to *M. tuberculosis* infection in humans remain very poorly understood. This is largely due to our inability to accurately discern disparate outcomes following exposure to *M. tuberculosis*. The TST and IGRAs measure the host response to antigens derived from *M. tuberculosis*, and their results have been used as a surrogate for infection. Epidemiologically, those with evidence of exposure to mycobacterial antigens are at much higher risk of progression to tuberculosis [66], although only a minority of individuals with positive test results develop disease. Continued negative results of TST or IGRAs following exposure to an infectious source case could be interpreted as indicating resistance to infection or a lack of true exposure. A major limitation in this regard has been the absence of animal models that reflect resistance, although studies performed in both cattle and rabbits have been promising [67, 68]. Another limitation has been tools that reflect true exposure, as many sources cases who have traditionally been thought to be infectious by virtue of having positive results of a sputum smear for acid-fast bacilli may not produce infectious aerosol [32].

Conceptually, resistance could be considered to reflect early clearance, exposure without evidence of adaptive immunity, or transient infection, as might be reflected in either reversion of TST or IGRA findings. Supporting the concept of early clearance are studies that have demonstrated the heritability of the TST response [69], as well as anecdotal reports of individuals who are repeatedly exposed to *M. tuberculosis* but continue to have negative TST or IGRA results. TST and IGRA test result reversions have been widely observed [70], and it is intriguing to note that, in a guinea pig model, reversion was associated with mycobacterial clearance following infection [71].

The well-known epidemiological correlates of vulnerability to tuberculosis are generally those promoting the development of disease once exposed to *M. tuberculosis*. In contrast, correlates of resistance to tuberculosis remain poorly understood. This is especially relevant to vaccine design, as the ideal vaccine would be one that prevents infection, rather than one that just diminishes the bacterial burden. In this regard, much of the vaccine modeling, either in mice, guinea pigs, or nonhuman primates, has not demonstrated prevention of infection and therefore does not yet provide evidence for a potential effect on tuberculosis transmission. Here, our current understanding of the mechanisms by which *M. tuberculosis* is controlled may not fully reflect the natural history that is seen in humans. In particular, the current paradigm of immune control of *M. tuberculosis* has centered on the granuloma and on the interaction of T cells with the infected cell. Specifically, it is widely believed that proinflammatory cytokines, such as interferon γ and tumor necrosis factor, can control intracellular growth of *M. tuberculosis* within macrophages through the production of NO and H_2_O_2_. However, recent evidence suggests that this model is incomplete. One challenge is that the macrophage is considered both the host and controller of *M. tuberculosis* [72].

Recent evidence, though, suggests that macrophages play a key role in the dissemination of *M. tuberculosis* [67, 73]. Further challenging this paradigm is recent work on the role of inducible NO synthase (iNOS) in the control of *M. tuberculosis*. Specifically, iNOS-deficient mice are vulnerable to infection with *M. tuberculosis*, supporting the model of NO-dependent control of *M. tuberculosis* replication [74]. However, this concept has been recently challenged by careful experiments in which mycobacterial growth can be distinguished from inflammation. Here, iNOS appears to regulate neutrophil-driven inflammation independently of the control of mycobacterial growth [75].

These data, then, leave open the question of how the immune system might contain infection with *M. tuberculosis*. One surprising finding is that cattle engineered to have enhanced macrophage expression of the TALE nickase are resistant to natural transmission of *M. tuberculosis* [68]. However, it is not clear how this might be translated into an improved vaccine. To address this, there are at least 3 possibilities. The first would be to improve macrophage function through the so-called training of stable epigenetic changes. Indeed, it has been proposed that this might be one way in which BCG vaccine can confer protection against nonmycobacterial diseases [76]. The second would be to develop a vaccine that elicits neutralizing, lung-resident antibodies. While available animal models have not supported this approach, none of them replicates prevention of infection. As a result, this concept is largely untested, and the role of antibodies in protection against tuberculosis remains controversial [77], although some recent data are very interesting in this respect [78]. The third possibility would be a vaccine that directs improved recognition of the infected cell in the lung. Because T cells can discern intracellular infection, this is an attractive possibility. In this regard, mucosal-associated invariant T (MAIT) cells are an innate-like T-cell subset prevalent in humans and enriched in the airway. Human MAIT cells have been defined by the expression of the semiinvariant T-cell receptor α chain TRAV1-2/TRAJ12/20/33 and their restriction by the nonpolymorphic major histocompatibility complex (MHC) class I–like molecule MHC-related protein 1. MAIT cells recognize *M. tuberculosis* and are activated by small organic molecules derived from the riboflavin biosynthesis pathway of bacteria and fungi [79]. Alternatively, aerosol delivery of conventional vaccines has the potential to generate a population of T cells capable of early recognition of *M. tuberculosis*–infected cells [80].

Ultimately, in considering vaccines or host-directed strategies to prevent infection with *M. tuberculosis*, modeling will be needed to evaluate novel concepts and approaches. These could be either improved animal models that reflect prevention of infection or human challenge models, as has been done for malaria. Because the mouse model continues to be a necessary first step in vaccine development, development of these models should be a high priority, possibly through the use of alternate mouse strains or in the generation of ultra-low-dose challenge models.

## OTHER CONSIDERATIONS

Central to the transmission of tuberculosis is *M. tuberculosis*, an organism that has evolved with its human host over millennia [81]. As already discussed in relation to drug-resistant strains, organism phenotype and lineage have been noted to be a determinant of virulence and transmissibility. The mechanisms through which this may arise are potentially numerous, possibly affecting every stage of transmission and disease in ways that are yet to be elucidated [44, 82].

Although *M. tuberculosis* is unique in its microbiology and immunopathology, some insight can be gained into the processes of respiratory pathogen transmission from studying viruses. Common features exist, both conceptual and mechanistic.

Respiratory viruses are generally thought to be transmitted by direct contact and large-droplet transmission, although there is controversy about the role of airborne transmission [83]. Mucosal immunity is likely to have direct impact on transmission by blocking or reducing viral shedding. Although serum neutralizing antibodies are the most widely studied and best understood correlates of protection, it is clear that systemic antibody levels often correlate poorly with susceptibility to infection or disease and that better correlates need to be identified, as with *M. tuberculosis* [84, 85]. The role of antibody in the susceptibility to *M. tuberculosis* and modulation of disease is controversial, and further investigation of mucosal responses is warranted [77].

The common anatomical site of *M. tuberculosis* and respiratory viral infections therefore leads to parallel dilemmas and potential approaches for further study. Recent studies of vaccine-induced and mucosal immunity [86] and of immunopathology in the context of experimental human influenza virus [87] and respiratory syncytial virus infections [88, 89], particularly in the lower airway, have broadened our understanding of the protective responses required to prevent infection, disease, and onward transmission. Similar developments in the *M. tuberculosis* field may therefore yield similar benefits.

Conversely, studies of tuberculosis transmission may also inform the epidemiology of other bacterial diseases. The cough aerosol sampling system first developed to collect infectious aerosols containing *M. tuberculosis* has been used to demonstrate the generation and transport of viable aerosols of *P. aeruginosa* and other gram-negative bacteria from persons with cystic fibrosis [47, 90]. These data suggest that understanding the modes and underlying mechanisms of various pathogens may have interdisciplinary benefit and could help better inform the scientific basis of infection control and public health policies.

## CONCLUSION

The transmission of tuberculosis is highly complex. Close consideration of the steps *M. tuberculosis* must take to leave one infectious individual and ultimately initiate infection and disease in a susceptible new host, as well as the many relevant variables that influence these steps, should lead to improved understanding of how transmission occurs and to increased chances for elimination of the disease.

## Notes

***Acknowledgments.*** We thank the workshop participants, for contributing to productive discussions relevant to this work; Drs N. Sarita Shah and Roxana Rustomjee, for their assistance in reviewing earlier versions of this manuscript; and the Division of AIDS, National Institute of Allergy and Infectious Diseases, for editorial and administrative assistance. Dr Fennelly’s effort was supported by the Intramural Research Program of the NIH, National Heart, Lung, and Blood Institute. We are grateful to Professor Bavesh Kana for contributing Figure 1 of this manuscript.

***Disclaimer.*** The views expressed in these manuscripts represent the authors’ opinions and not necessarily represent that of the National Institute of Allergy and Infectious Diseases or National Institutes of Health.

***Financial support.*** This work was supported by the Division of AIDS, National Institute of Allergy and Infectious Disease, National Institute of Health, Department of Health and Human Services (contract HHSN272201100001G [Research Support Services for the Division of AIDS]), the Bill and Melinda Gates Foundation, the Centers for Disease Control and Prevention, and the South African Medical Research Council.

***Supplement sponsorship.*** This work is part of a supplement sponsored by the Centers for Disease Control and Prevention and the National Institutes of Health.

***Potential conflicts of interest.*** All authors: No reported conflicts of interest. All authors have submitted the ICMJE Form for Disclosure of Potential Conflicts of Interest. Conflicts that the editors consider relevant to the content of the manuscript have been disclosed.
